# Understanding Challenges of Recruiting African Americans for Genomic Studies in Alzheimer’s Disease Beyond Trust

**DOI:** 10.1002/alz.093302

**Published:** 2025-01-09

**Authors:** Takiyah D. Starks, Allison M Caban‐Holt, Kristy A. Terrell, Jesseca Crawford, Ashley Gregory, Larry D. Adams, Michael L. Cuccaro, Jonathan L. Haines, Christiane Reitz, Jeffery M. Vance, Margaret A Pericak‐Vance, Goldie S. Byrd

**Affiliations:** ^1^ Maya Angelou Center for Health Equity, Wake Forest University School of Medicine, Winston‐Salem, NC USA; ^2^ Maya Angelou Center for Health Equity, Winston‐Salem, NC USA; ^3^ Maya Angelou Center for Health Equity at Wake Forest University School of Medicine, Winston‐Salem, NC USA; ^4^ John P. Hussman Institute for Human Genomics, Miami, FL USA; ^5^ John P. Hussman Institute for Human Genomics, University of Miami Miller School of Medicine, Miami, FL USA; ^6^ Department of Population and Quantitative Health Sciences, Institute for Computational Biology, Case Western Reserve University, Cleveland, OH USA; ^7^ Gertrude H. Sergievsky Center, Taub Institute for Research on the Aging Brain, Departments of Neurology, Psychiatry, and Epidemiology, College of Physicians and Surgeons, Columbia University, New York, NY USA; ^8^ Dr. John T. Macdonald Foundation Department of Human Genetics, University of Miami Miller School of Medicine, Miami, FL USA

## Abstract

**Background:**

Black/African Americans (B/AAs) remain underrepresented in Alzheimer’s disease (AD) research and clinical trials. As a part of understanding genetics and genomics of AD, four U.S sites are recruiting 4,000 African Americans over age 60 for research participation. We identify challenges and propose solutions for recruiting AA’s using community based and grass roots efforts.

**Method:**

An important strategy for recruiting B/AAs for research is to engage researchers and staff in face‐to‐face interactions outside of the medical/academic campus. We analyzed steps required to move individuals from initial research interest to their enrolling in the study, over a 6‐month period. During community outreach activities, interested individuals provided basic demographic data, and level of interest in AD research. Research staff followed‐up by telephone up to three times, within 48 ‐72 hours, to answer questions and schedule a home visit. Scheduled participants were also contacted one week before the study appointment, and a confirmation letter was sent. Participants were called the day before and the day of the home visit to assure participant availability, creating up to eight touch points prior to enrollment.

**Result:**

We enrolled 67 of the 129 B/AAs scheduled over a 6‐month period. Thirty three of the 67 interested participants (49%) were scheduled on the first attempt, n = 26 (39%) on the second attempt and n = 8 (12%) on the third attempt. All enrollees (100%) were called a week before the appointment and a confirmation email was sent. Thirteen (10%) of scheduled appointments were cancelled due to: 1) inability to reach them (62%), 2) no longer interested (15%), 3) feared the blood draw (15%) and 4) time constraints (8%).

**Conclusion:**

Despite using culturally sensitive research teams, materials and advertisements, reliability of recruiting B/AAs for genetic research studies using grass roots methods remains elusive. Appointment cancellations due to fear of blood draws, family issues and time limitations are key factors, but understanding reasons for loss of contact and interest should be further investigated. Closing gaps between B/AAs’ research interest and their actual enrollment will require multiple and simultaneous recruitment strategies that might require more than trust.

